# Sensitization of ovarian cancer cells to cisplatin by gold nanoparticles

**DOI:** 10.18632/oncotarget.2203

**Published:** 2014-07-11

**Authors:** Xunhao Xiong, Rochelle R. Arvizo, Sounik Saha, David J. Robertson, Scott McMeekin, Resham Bhattacharya, Priyabrata Mukherjee

**Affiliations:** ^1^ Department of Pathology, The University of Oklahoma Health Sciences Center, Oklahoma City, Oklahoma; ^2^ Department of Physiology and Biomedical Engineering, Mayo Clinic College of Medicine, Rochester, MN; ^3^ Department of Chemistry and University of Missouri Research Reactor, University of Missouri, Columbia, Missouri; ^4^ Obstetrics and Gynecology, The University of Oklahoma Health Sciences Center, Oklahoma City, Oklahoma; ^5^ Peggy and Charles Stephenson Cancer Center, The University of Oklahoma Health Sciences Center, Oklahoma City, Oklahoma

**Keywords:** gold nanoparticle, chemoresistance, cancer stem cell, EMT, NF-κB

## Abstract

Recently we reported that gold nanoparticles (AuNPs) inhibit ovarian tumor growth and metastasis in mice by reversing epithelial-mesenchymal transition (EMT). Since EMT is known to confer drug resistance to cancer cells, we wanted to investigate whether anti-EMT property of AuNP could be utilized to sensitize ovarian cancer cells to cisplatin. Herein, we report that AuNPs prevent cisplatin-induced acquired chemoresistance and stemness in ovarian cancer cells and sensitize them to cisplatin. AuNPs inhibit cisplatin induced EMT, decrease the side population cells and key stem cell markers such as ALDH1, CD44, CD133, Sox2, MDR1 and ABCG2 in ovarian cancer cells. Mechanistically, AuNPs prevent cisplatin-induced activation of Akt and NF-κB signaling axis in ovarian cancer cells that are critical for EMT, stem cell maintenance and drug resistance. *In vivo*, AuNPs sensitize orthotopically implanted ovarian tumor to a low dose of cisplatin and significantly inhibit tumor growth via facilitated delivery of both AuNP and cisplatin. These findings suggest that by depleting stem cell pools and inhibiting key molecular pathways gold nanoparticles sensitize ovarian cancer cells to cisplatin and may be used in combination to inhibit tumor growth and metastasis in ovarian cancer.

## INTRODUCTION

Epithelial ovarian cancer (EOC) is one of the deadliest gynecological malignancies of women in the western world. Primary management includes an extensive surgical debulking followed by combination chemotherapy including platinum and taxane based regimen. Despite frequent initial responses indicating sensitivity to platinum agent, most patients with advanced stage disease eventually develop platinum resistance which leads to low responsiveness to any agents and shortened survival [1,2]. Therefore, overcoming platinum resistance is an urgent need in the therapeutic management of EOC.

Early efforts have demonstrated that epithelial-mesenchymal transition (EMT) plays critical roles in ovarian cancer progression including increasing migration and invasion ability [3,4], contributing to chemoresistance acquisition [5,6] which could give rise to recurrence and metastasis after standard chemotherapeutic treatment. In addition, EMT and acquisition of chemoresistance are also believed to correlate with “cancer stem-like cells” which represent the most tumorigenic and treatment-resistant cells within a heterogeneous tumor mass [7-10]. Interestingly, Yew and colleagues recently reported that epimorphin reverted ovarian cancer cells away from a mesenchymal phenotype toward an epithelial phenotype, thereby enhancing sensitivity to carboplatin [11]. Therefore, targeting EMT offers an attractive therapeutic option for overcoming drug resistance in ovarian cancer patients.

Over the past decade, nanotechnology has received considerable attention for cancer therapy [12-14]. It provides a unique approach and comprehensive technology against cancer because of the special optical, magnetic, or structural properties of the nanometer-sized particles [15-19]. Our group has demonstrated that 20 nm gold nanoparticles (AuNPs) inhibited proliferation, angiogenesis and metastases in a preclinical mouse model of ovarian cancer [12,20,21]. At the molecular level, treatment with AuNPs altered the profiles of a series of secretory cytokines [18], a majority of which are key elements in regulating signaling related to EMT and stem cell maintenance. Owing to these unique and remarkable properties, we postulated that a low dose of AuNPs could be utilized to sensitize ovarian tumors to chemotherapy such as cisplatin. Herein, we show that pretreatment with AuNPs prevented cisplatin-induced chemoresistance acquisition by depleting the stem cell pool, downregulating multidrug resistance gene and inhibiting NF-κB/Akt signaling. The present work provides a new therapeutic strategy to intervene in relapsed and refractory ovarian cancer cases, therapeutic management of which is still an overwhelming challenge.

## RESULTS

### AuNPs sensitize cancer cells to chemotherapeutics *in vitro*

**Figure 1:**
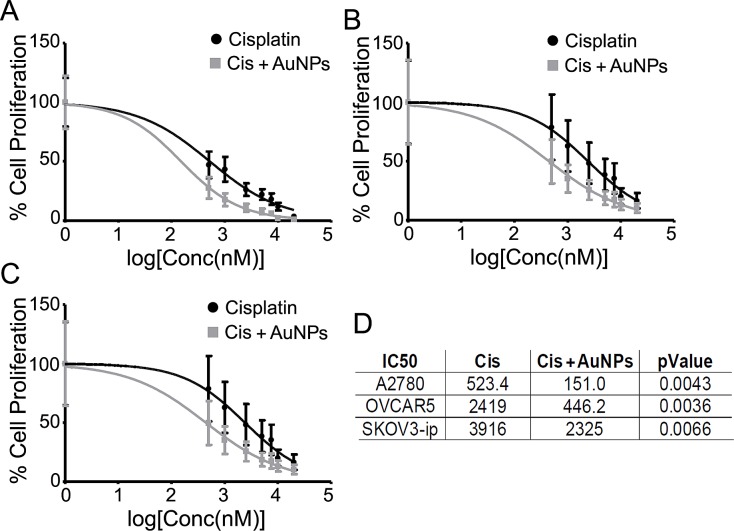
AuNPs sensitize ovarian cancer cells to cisplatin Serum starved A2780 (A), OVCAR5 (B) and SKOV3-ip (C) were initially treated with (Cis+AuNPs; grey line) or without (Cis only; black line) 5 μg/ml of 20 nm AuNP for 24 hours and then exposed to various concentrations of cisplatin for another 24 hours. IC_50_ values were determined by [^3^H]-thymidine incorporation assay. (D) Comparison of IC_50_ values for cisplatin with and without AuNPs against ovarian cancer cells A2780, OVCAR5 and SKOV3-ip. Values are means ± SD. N=3.

We previously demonstrated that AuNPs exhibited an anti-tumorigenic effect in ovarian cancer in a size and dose dependent manner [12,20] and reversed EMT. Since EMT plays a critical role in drug resistance, we wanted to investigate whether AuNPs could sensitize ovarian cancer cells to cisplatin. After 24 hours exposure to a low dose of 20 nm AuNPs (5μg) (physicochemical characterization is provided in Fig. S1), ovarian cancer cells were treated with various concentration of cisplatin for an additional 24 hours. Significant reduction (2-5 fold) in the 50% inhibitory concentration (IC_50_) of cisplatin was observed in AuNP pretreated A2780, OVCAR5 and SKOV3-ip cells (Fig. 1 A-D), indicating that treatment with AuNPs significantly sensitize ovarian cancer cells to cisplatin.

### AuNP prevents cisplatin-induced EMT

**Figure 2:**
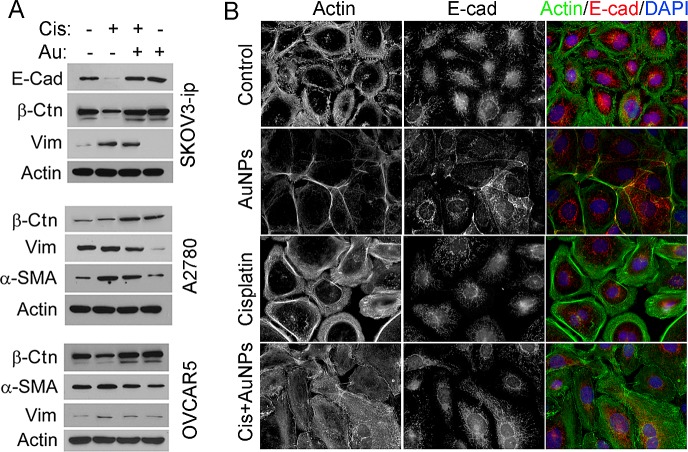
AuNP reverses cisplatin-induced EMT Serum starved A2780, OVCAR5 and SKOV3-ip were treated with 20 nm AuNP for 24 hours (Au), cisplatin for 72 hours (Cis), or pretreated with AuNPs followed by cisplatin treatment (Cis + AuNPs), untreated cells were used as control. (A) The cell lysates were immunoblotted with antibodies for selected EMT markers. Actin was used as a loading control. Vim, Vimentin; β-Ctn, β-Catenin. (B) The cells morphology changed after cisplatin and/or AuNPs treatment. The fixed cells were stained using Alexa Fluor 488-Phalloidin (1:500) and Anti-E-cadherin antibody (1:500) followed by Alexa Fluor 568-conjugated secondary antibody, respectively. Then the localization of E-cadherin and F-actin were visualized by immunofluorescence microscopy.

EMT is one of the main mechanisms underlying development of tumor growth and metastasis, which induces stem-like properties and confers drug resistance to tumor cells [22,23]. Previously, we reported that AuNPs reversed EMT both *in vitro* and *in vivo*, therefore, we hypothesized that pretreatment with AuNPs could inhibit cisplatin-induced EMT, a process critically required for chemoresistance acquisition. We next determined the expression profile of several EMT markers in various ovarian cancer cell lines with or without cisplatin and AuNP treatment. Incubation with cisplatin led to a significant up-regulation of mesenchymal markers such as vimentin and α-smooth muscle actin (α-SMA) and down-regulation of epithelial markers such as E-cadherin and/or β-Catenin simultaneously (Fig. 2 A). In addition, pretreatment with AuNPs significantly blunted the EMT-inducing effect of cisplatin by decreasing expression of EMT markers (Fig. 2 A). To observe this morphological transition, E-cadherin and F-actin in SKOV3-ip cells was visualized by immunofluorescence. Both F-Actin and E-Cadherin were recruited to cellular junctions from cytosol after being treated by AuNP alone (Fig. 2 B). Interestingly, cisplatin treatment disrupted filopodia-like cell-cell contacts as visualized by F-actin staining, supporting induction of a mesenchymal phenotype. Importantly, pretreatment with AuNPs prevented cisplatin-induced EMT and exhibited up-regulation of E-Cadherin/ β-Catenin and down-regulation of vimentin and α-SMA compared to the cells treated by cisplatin alone, although most of the E-cadherin was still localized in the cytosol (Fig. 2 A and B).

### AuNP suppresses cancer stem cell properties

**Figure 3:**
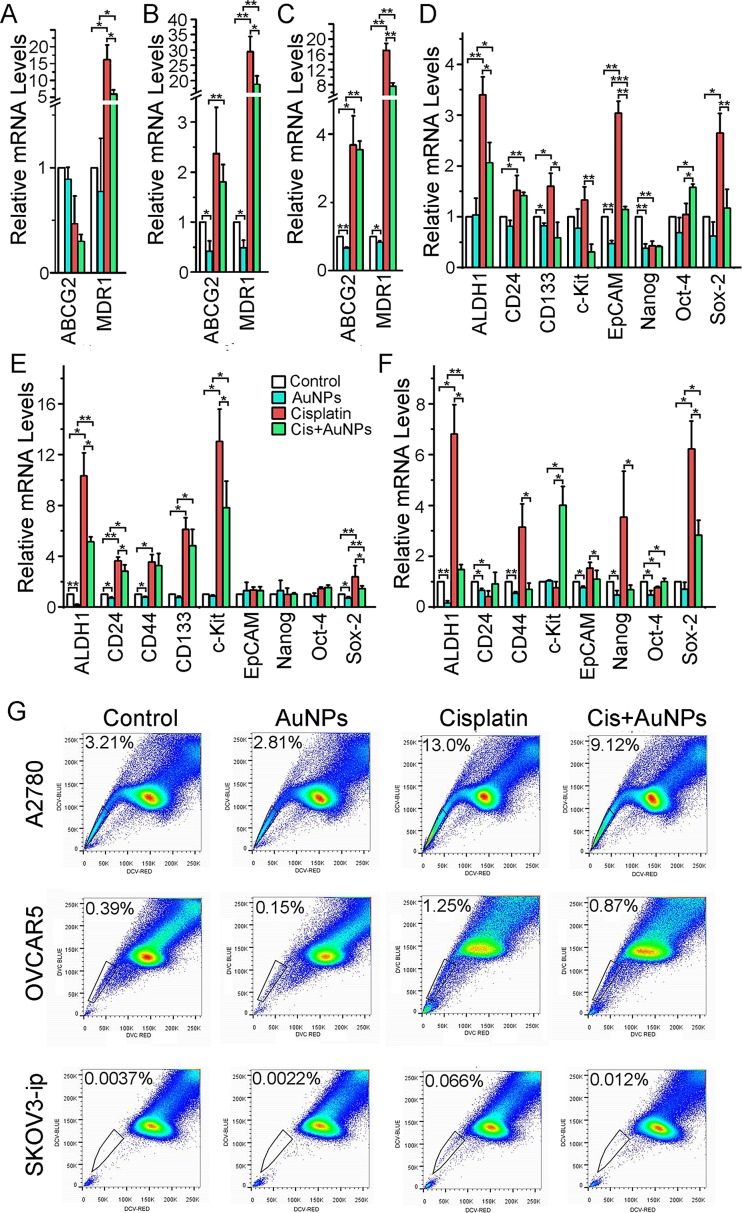
AuNP dowregulates the expression of key stem cell markers and the side population of ovarian cancer cells Serum starved A2780 (A, D), OVCAR5 (B, E) and SKOV3-ip (C, F) were treated as Fig. 2 above, respectively. (A-F) The cisplatin and/or AuNPs treatment altered the expression of selected stem cell markers. The total RNAs were extracted from the treated/untreated cells and subjected RT-PCR; Relative quantification of target genes was calculated using the comparative cycle threshold (CT) method (2^−ΔΔCT^) with genes normalized to GAPDH. Values are means ±SD. *P < 0.05, **P < 0.01, ***P<0.001. N=3. (G) AuNPs down-regulated the cisplatin-induced side population cells. The indicated cells were labeled with DyeCycle Violet and the side population cells were counted by flow cytometry.

Acquired chemoresistance in ovarian cancer is associated with EMT and a more cancer stem cell-like (CSC) phenotype [7,9]. Therefore, we investigated whether AuNPs prevented cisplatin-induced enrichment of CSC pools, and if enrichment of cellular stemness could be a plausible mechanism for acquired chemoresistance in ovarian cancer cells. Up-regulation of MDR1 and ABCG2 expression is one of the hallmarks of acquired chemoresistance. As seen in Fig. 3 A-C, treatment with cisplatin dramatically increased the expression of MDR1. This increase was however inhibited upon pretreatment of the cells with AuNP. To gain deeper insight into the induction of stemness by cisplatin and the effect of AuNP pretreatment, we conducted quantitative RT-PCR experiments for a few candidate stem cell markers [8,24,25] in ovarian cancer cells. The results showed that pretreatment with AuNPs significantly prevented cisplatin-induced up-regulation of a number of key stem-cell markers, including ALDH1, CD133, EpCAM, c-Kit and Sox-2 in A2780 cells; ALDH1, CD24, c-Kit and Sox-2 in OVCAR5 cells; and ALDH1, CD44, EpCAM and Sox-2 in SKOV3-ip cells (Fig. 3 D-F). These results suggest that AuNPs may potentially prevent cisplatin-induced acquired stemness in ovarian cancer cells.

Next we sought to investigate whether prevention of cisplatin-induced stemness by AuNP actually leads to the depletion of stem cell like pools by analyzing the side population of ovarian cancer cells after only cisplatin treatment and cisplatin treatment after AuNP pretreatment. It is long known that the side population (SP) of ovarian cancer cells engender stem-like characteristics [26] including chemoresistance [7,9]. Therefore, the cells were stained with DyeCycle Violet dye and the stem-like cells were evaluated by side population analysis. According to Fig. 3 G, there is a marked increase in the percentage of SP cells upon treatment of A2780 cells with cisplatin. While the non-treated A2780 control cells have ~ 3% SP cells, treatment with cisplatin enriched the SP cell pool to ~13%. Importantly, pretreatment with AuNPs prevented cisplatin-induced enrichment of SP cells and reduced it to ~9%. A similar trend was also observed with OVCAR5 and SKOV3-ip cells. Taken together, these results demonstrated that pretreatment with AuNPs reduced cisplatin-induced acquired ‘stemness’ and enrichment of CSC like SP cells in ovarian cancer, which may be the mechanism of cisplatin sensitization by AuNP.

### AuNP inhibits the activation of Akt/NF-κB signaling

**Figure 4:**
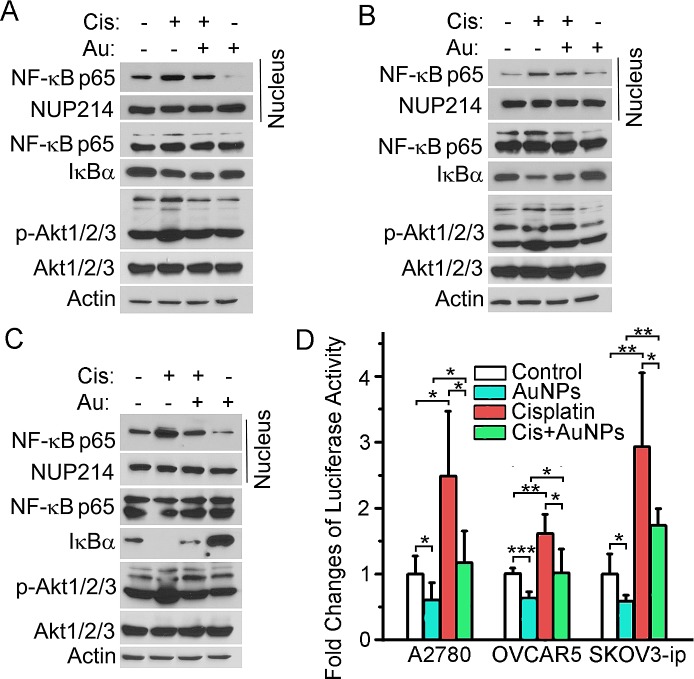
AuNPs inhibit the Akt/NF-κB signaling Serum starved A2780 (A), OVCAR5 (B) and SKOV3-ip (C) were treated as Fig. 2 above. The indicated protein levels in total lysates were analyzed by western blotting and actin was used as loading control. Nuclear protein extracts of cells were also obtained and subjected to western blotting to evaluate the nuclear NF-κB p65 level. NUP214 (nucleoporin 214 kDa) was used as the loading control for nuclear protein. (D) AuNPs inhibit the NF-κB-dependent transcription. NF-κB-luciferase transfected A2780, OVCAR5 and SKOV3-ip were treated as above, and the NF-κB activation was evaluated by detecting the luciferase activity. N=3. Values are means ±SD. *P < 0.05, **P < 0.01, ***P<0.001.

The Akt/NF-κB signaling axis is critical in regulating cell survival, inflammation, EMT, CSCs as well as chemoresistance [23,27-30]. In addition, NF-κB activation by cisplatin has been reported in various cancers [31-33]. Moreover, salinomycin, an eliminator of CSCs, inhibited Akt/NF-κB signaling in cisplatin-resistant ovarian cancer cells [34]. Hence perturbation of the Akt/NF-κB pathway by AuNP in cisplatin-induced cells could explain the loss of acquired chemoresistance and ‘stemness’ in ovarian cancer cells. It is evident from Fig. 4 A-C that incubation with cisplatin prompted degradation of IκBα in ovarian cancer cells with a concomitant increase in NF-κB p65 levels in the nuclear fraction (Fig. 4 A-C). In agreement with western blotting, cisplatin treatment increased NF-κB-luciferase activity by 50% in OVCAR5, and more than 150% in A2780 and SKOV3-ip cells (Fig. 4 D). However, pretreatment with AuNPs prevented cisplatin-induced degradation of IκBα and thus an increase in IκBα levels were observed upon AuNP pretreatment (Fig. 4 A-C). Consequently, AuNP-pretreatment also led to a decrease in NF-κB p65 levels in the nuclear fraction (Fig. 4 A-C). A significant decrease in luciferase activity was observed in all the three cell lines, further confirming the prevention of cisplatin-induced NF-κB activation upon AuNP-pretreatment (Fig. 4 D). Therefore, NF-κB signaling might be involved in AuNP-mediated sensitization of ovarian cancer cells to cisplatin.

Akt can activate NF-κB via regulating I-κB kinase (IKK), and can cross-talk with MAPK resulting in transcription of pro-survival genes. Our previous work demonstrated that AuNPs inhibited ovarian cancer cells proliferation and tumor growth by abrogating MAPK-signaling [20]. Therefore, we determined whether the Akt signaling was regulated by cisplatin and/or AuNPs in ovarian cancer cells. According to Fig. 4 A-C, cisplatin significantly induced activation of Akt although the total Akt levels did not change. Mechanistically, AuNPs treatment decreased cisplatin-induced Akt activation. Taken together, AuNP induced sensitization of cisplatin to ovarian cancer cells is most likely through inhibition of the Akt/NF-κB signaling axis.

### AuNP enhances cisplatin sensitivity *in vivo*

**Figure 5:**
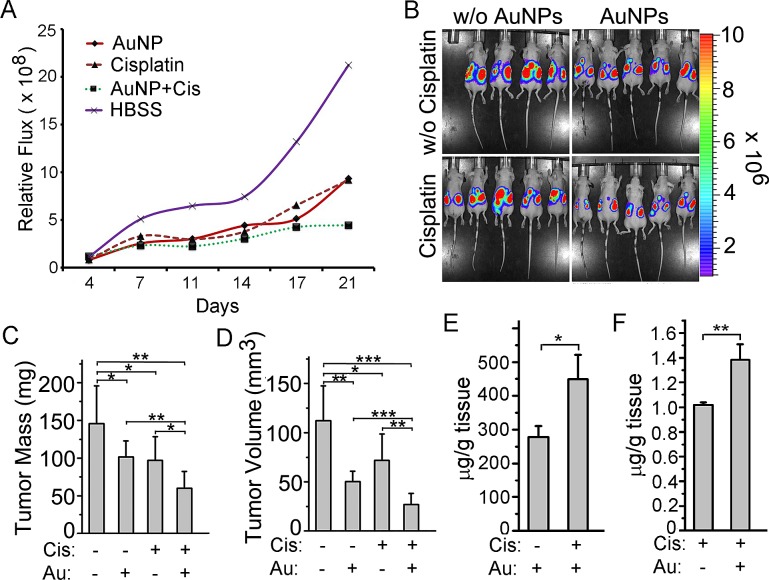
AuNPs combined with cisplatin treatment reduces tumor growth in a mouse model of ovarian cancer (A) Tumor growth and metastasis were monitored by non-invasive bioluminescence analysis over three weeks using a Xenogen-IVIS–cooled CCD optical system. Mice treated with AuNPs and cisplatin show a diminution in flux comparatively. (B) Bioluminescence image on day 21 depicting tumor presence in the different groups. (C, D) Statistical analysis of final tumor mass (C) and volume (D) of the mice tumors shows that combined treatment by AuNPs and cisplatin significantly decrease the tumor growth. (E, F) Combined treatment enhances the uptake of both AuNPs and cisplatin. Tumor tissues were analyzed for gold uptake uisng INAA (E) and cisplatin uptake using ICP-MS (F). Each group contains 9~10 mice. Values are means ±SD. *P < 0.05, **P <0.01, ***P<0.001.

Since AuNP lowered the IC_50_ value of cisplatin and pruned ovarian cancer cells towards chemosensitization by depleting stem cell pools and inhibiting NF-κB/Akt signaling axis, we wanted to investigate whether a low dose of cisplatin could be used to effectively inhibit ovarian tumor growth in AuNP treated animals. We implanted SKOV3-ip-luc cells intrabursally into the ovaries (6–8-wk-old athymic nude female mice) and monitored the tumor growth and metastasis noninvasively using bioluminescence over time. For 3 weeks the mice received i.p. AuNP (100 μg) or HBSS at 3 dose/wk and cisplatin at 500 μg/kg body weight or HBSS on alternate days. On day 22, the mice were euthanized and the tumors and nodules were collected for further analysis. A notable decrease in bioluminescence of the groups treated with AuNP or cisplatin (Cis) compared to the HBSS control group (Fig. 5 A) was noted. However, the group pretreated with AuNPs followed by cisplatin (Cis+AuNPs) showed the highest growth retardation of the tumors and the weakest bioluminescence signal (Fig. 5 A and B). The health of the mice in each group was monitored daily. As shown in Fig. S2 A, the weight gain of the mice is typical over the study period, indicating that the dose schedule for each group was non-toxic.

The regression in tumor growth was confirmed by measuring the tumor mass and volume at the end of the study (Fig. 5 C and D, Fig. S2 and S3). The Cis+AuNPs group showed the tightest cluster of data points as well as the highest therapeutic effect among all the groups (Fig. S2 B and C). Uptake of AuNP and cisplatin showed about 50% increase in the Cis+AuNPs group (Fig. 5 E and F). Moreover, nodule mass and volume had also decreased and a 50% decrease in nodule formation was noticed compared to the HBSS group (P<0.001; Fig. S2 B-D).

**Figure 6:**
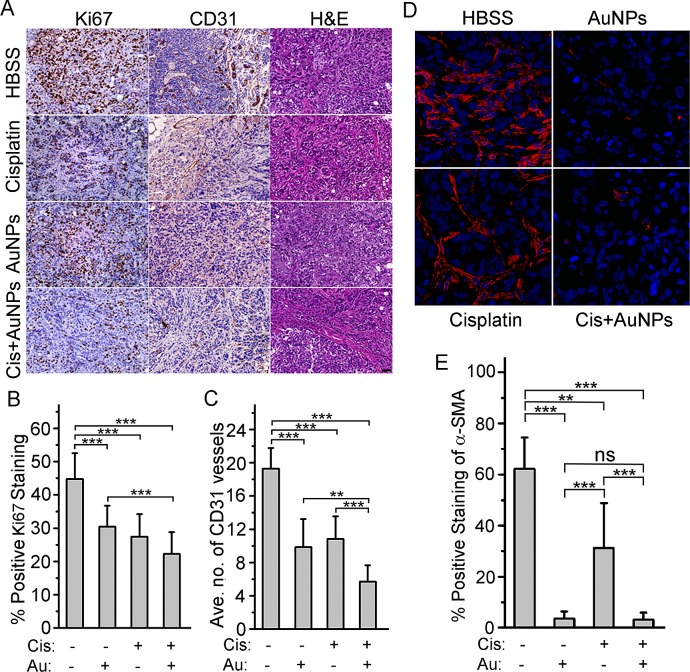
AuNP treatment inhibits tumor cells proliferation, blood vessels formation and EMT *in vivo* (A) Representative histology of tumors from mice xenografts of SKOV3-ip cells with Ki67 and CD31 expression. Images were taken at a 20x magnification. (B) Image analysis of Ki67 staining shows a notable reduction in all treated samples compared to the HBSS treated group. A substantial reduction is also seen between the nanoparticle only (AuNP) and the nanoparticle with cisplatin (Cis+AuNPs) groups (C) Image analysis of CD31 staining analysis showed a remarkable reduction in vessel formation in treated samples compared to the HBSS treated group. The Cis+AuNPs group showed a further reduction in vessel formation compared to the groups treated with nanoparticle (AuNP) or cisplatin (Cis) only. Each group contains 9~10 mice. (D) Immunohistochemistry / immunofluorescence staining of mice tumor tissues with α-SMA antibody. (E) Image analysis of α-SMA staining showed a remarkable reduction of myofibroblasts in AuNPs treated groups. N=4. Values are means ±SD. *ns =* not significant, *P < 0.05, **P < 0.01, ***P<0.001

Growth regression was further confirmed by quantifying the number of proliferating cells using Ki67 staining (Fig. 6 A and B). A significant decrease in Ki-67 staining was observed in the treatment groups as compared to the control HBSS group. However, no significant difference between the single therapy treated groups (AuNP vs. Cis) and the two groups treated with cisplatin (Cis vs. Cis+AuNPs) was observed. Interestingly, there was a remarkable difference (P<0.001) between the nanoparticle treated groups (AuNP vs. Cis+AuNPs). Priming the tumors with 20 nm AuNPs may allow for normalization and remodeling of the tumor vasculature thus permitting small molecules, such as cisplatin, to be effectively delivered [17]. To evaluate this, CD31 staining was performed. There was a 50% decrease of cells positive for CD31 in the single therapy groups (AuNP and Cis) compared to the HBSS treated group and 75% decrease in cells positive for CD31 in the Cis+AuNPs group (Fig. 6 A and C). Remarkably, there is a 50% difference between the AuNP+Cis group and the single therapy groups with the biggest significance being between Cis+AuNPs and the Cis only group (P<0.001). All of this data convincingly demonstrated that AuNPs sensitized ovarian cancer cells to cisplatin both *in vitro* and *in vivo*.

To identify epithelial cells undergoing EMT *in vivo*, xenograft tumor tissues from mice were immunostained for the mesenchymal marker α-SMA. This antibody recognizes both myofibroblasts and blood vessels (Fig. 6 D, Fig. S4). Alpha-SMA stained blood vessels depicted a distinct staining pattern (Fig. S4) and were excluded from quantitation in order to evaluate EMT. The percentage of α-SMA positive fibroblast-like cells significantly decreased in the AuNP treated group (Fig. 6 D and E). Although the tumor growth was comparable in the Cisplatin and AuNP group (Fig. 5 C and D), the percentage of α-SMA positive fibroblast-like cells was significantly lower in the AuNP group (Fig. 6 D and E). Together, these data suggest that pretreatment with AuNP decreased the efficacy of cisplatin-induced EMT in xenograft ovarian tumors *in vivo*.

## DISCUSSION

Since 1980 cisplatin and its analogues have remained the backbone of systemic therapy for EOC with recent advances being a change in the mode of delivery to intraperitoneal. Although ovarian cancer is among the most chemosensitive malignancies at the time of initial treatment by surgery and taxane/platinum-based therapy, most patients ultimately relapse and succumb to chemoresistant disease [1,2,35]. Furthermore, front line chemotherapy is associated with toxicities and may adversely impact the quality of life [36]. Given this disappointing situation, our aim was to first test if AuNPs could enhance the efficacy of cisplatin by sensitizing EOC and secondly to investigate the underlying molecular mechanisms.

Several lines of evidence suggest that the heterogeneous cancer cells employ a dynamic survival strategy in which a small subpopulation assume a reversible drug-tolerant state that can protect the population from eradication by potentially lethal exposures [37]. EMT reflects such an adaptation conferring stem-like properties to cancer cells, leading to resistance to cytotoxic drugs and metastasis [3-6,20,38]. Since AuNP treatment reversed EMT in cancer cells by reducing secretion of TGF-β, bFGF and uPA, proteins involved in EMT, up-regulating E-Cadherin, and down-regulating Snail, N-Cadherin, and Vimentin both *in vitro* and *in vivo* [20], we hypothesized that AuNPs can potentially act as a multifunctional molecule to increase sensitivity of cells towards cisplatin.

In this study, we demonstrate that AuNPs sensitized cancer cells to cisplatin *in vitro* and *in vivo*. In order to understand a role of AuNP in pruning cancer cells to cisplatin, we utilized a low concentration of AuNP (5 μg/ml) based on our previous study that resulted in a modest 10-15% inhibition of proliferation of ovarian cancer cell lines [20]. Among the three cell lines tested for *in vitro* studies, we selected SKOV3-ip cells for the *in vivo* study because they demonstrated lowest sensitization in *in vitro* studies. Also, our previous study showed that SKOV3-ip cells metastasized into the peritoneal cavity after orthotopic implantation into the ovarian bursa and an intraperitoneal administration of 200 μg of AuNP per animal inhibited tumor growth and metastasis [20]. Therefore, in this work we decided to use a low dose, 100 μg of AuNP/animal/treatment, to determine a role in cisplatin sensitization *in vivo*. At clinically relevant concentrations cisplatin mediated nephrotoxicity is a major stumbling block in therapy [39]. For this reason we utilized cisplatin at 500μg/kg/dose, a concentration about 10 times lower than usual preclinical doses. We demonstrated that pretreatment with AuNPs enhanced cisplatin efficacy both *in vitro* and *in vivo*. In addition, uptake of AuNPs and cisplatin were observed to be increased in the AuNP and cisplatin combination group (Fig. 5 E and F). Therefore, AuNPs might be used clinically with lower dose cisplatin thus reducing the nephrotoxicity.

Most interestingly, we show that cisplatin treatment downregulated epithelial markers such as E-Cadherin and β-Catenin, and upregulated mesenchymal markers such as vimentin and α-SMA, implying that an EMT-like phenotype was induced by cisplatin, which is in accordance with recent reports [20]. However, pretreatment with AuNPs attenuated this EMT process. Taken together, these results suggest that at least one of the mechanisms by which AuNPs sensitize the cells to cisplatin might be through reversal of EMT.

Since EMT has the potential to confer stem-like properties to a subpopulation of cancer cells that would be resistant to chemotherapeutics, we next tested whether the resistance to cisplatin correlated with CSCs in ovarian cancer cells. Cisplatin treatment upregulated the expression of several stem cell markers such as ALDH1, CD24, CD44, CD133, EpCAM, Nanog, Oct-4 and Sox2, and increased the SP simultaneously. Also pretreatment with AuNPs prevented cisplatin-induced acquired ‘stemness’ and enrichment of SP cells. To our knowledge, our data for the first time demonstrated that AuNP inhibited the expression of stem cell markers and reduced the pool of side population cells. Exploring the signaling that regulates AuNP-mediated inhibition of stemness will help to identify key players involved in the cisplatin resistant and develop AuNP-based combination therapy. Recent reports demonstrated that nanoparticles could induced endoplasmic reticulum (ER) stress and reactive oxygen species (ROS) production [40,41]. Interestingly, several groups reported that endoplasmic reticulum (ER) stress led to the loss of epithelial stemness [42,43]. Furthermore, ROS has also been implicated in chemoresistance in various cancers [44,45]. Therefore, it is possible that AuNPs might inhibit epithelial stemness through the induction of ER stress.

The Akt/NF-κB signaling axis is critical in regulating cell survival, EMT, CSCs as well as resistance to chemotherapeutics. Reports from several groups have highlighted the potential use of combination therapy involving cisplatin and Akt/NF-κB inhibitors [31,33]. Recently salinomycin, an eliminator of CSCs, has been demonstrated to inhibit Akt/NF-κB in cisplatin resistant ovarian cancer cells [34]. Here, we demonstrate that cisplatin treatment activated Akt/NF-κB signaling in ovarian cancer cells and pretreatment with AuNPs attenuated this effect. Therefore, inhibition of Akt/NF-κB signaling by AuNPs provides an inorganic nanomaterial based therapeutic approach for sensitizing cells to cisplatin by decreasing EMT and stemness and thus may play an important role in therapeutic management of ovarian cancer. In this context activation of the Akt/NF-κB signaling could be due to activation of survival pathways post cisplatin stress or due to enrichment of the growth factor signaling in stem cell populations, as demonstrated for TGF-β, bFGF and uPA that AuNP inhibits [20].

After decades of efforts, mechanisms underlying cisplatin resistance has been considered to be multifactorial that includes changes in drug transport, DNA repair and damage as well as alterations in cell death / apoptosis pathways [46]. One major problem for overcoming this clinically relevant issue is that more than one resistance mechanism is activated. In view of these considerations, it might be a more successful strategy for circumventing resistance by targeting multiple mechanisms. The distinct properties of AuNP in antiangiogenesis, reversing EMT, inhibiting “stemness”, and enhancing cisplatin uptake make it as an efficient candidate for overcoming chemoresistance. Because of the low toxicity, gold-based compounds have long been used as anti-inflammatory agents to treat rheumatoid arthritis [47]. Therefore, gold nanoparticles may potentially alleviate the side effects of cisplatin and be used in combination to inhibit ovarian tumor growth and metastasis in the clinic.

Although a promising strategy, the use of cisplatin and AuNPs combination for anticancer therapy still faces some important challenges. First, more pre-clinical studies are still required to assess the safety of nanoparticles at the whole animal level, *in vivo*. Second, as the combination treatment increases the uptake of both AuNPs and cisplatin, therapeutic agents are able to reach the targeted area as well as normal tissues. To avoid unwanted toxicity, optimizing the mode of administration and drug dosage may be necessary. Similar to pharmaceutical drugs, studying the pharmacokinetics of nanoparticles *in vivo* to assess their absorption, biodistribution, metabolism, elimination processes is essential. In addition, specific tissue-level toxicological studies are also required, which include the hepatotoxicity (liver), nephrotoxicity (kidney), immunogenicity, hematological toxicity (blood), and inflammatory and oxidative responses due to the nanoparticles.

In summary, we demonstrate here that exposure to exogenous AuNP is capable of inducing an epithelial-like phenotype in the ovarian cancer cells exhibiting mesenchymal features. Pruning the cells with AuNP prevents enrichment of stem cell pools, reduces expression of multidrug resistance genes and inhibits critical signaling pathways required for stem cell maintenance, EMT and drug resistance. Thus, the present report supports that gold nanoparticle performs as a molecular ‘brake’ that prevents cisplatin induced ‘run-away’ activation of Akt/NF-κB pathways leading to acquired stemness and drug resistance phenotype. The property of AuNPs to sensitize ovarian cancer cells to a low dose cisplatin may alleviate the potential dose limiting toxicity and extend the therapeutic application in a broad range of cancers that warrants further clinical investigation.

## MATERIALS AND METHODS

### Chemical Reagents and Antibodies

Tetrachloroauric acid trihydrate, trisodium citrate and sodium borohydride were from Sigma-Aldrich, St. Louis, MO. [^3^H] Thymidine was from Perkin-Elmer, (Waltham, MA). Media and PBS was purchased from Mediatech (Manassas, VA). Cisplatin was obtained from the Mayo Clinic Pharmacy services at a concentration of 50mg/ml. Scintillation cocktail was purchased through Fisher Scientific. And Alexa Fluor® 488 Phalloidin is from Life Technologies.

The following antibodies were used for Western blotting and immunofluorescence: anti–E-cadherin, anti-N-Cadherin, anti-β-Catenin, and anti-vimentin (BD Biosciences); anti-α-SMA, anti-Ki67, and anti-β-actin (Sigma-Aldrich); anti-IκBα and anti-p65 (Cell Signaling Technology); anti-CD31, anti-AKT1/2/3, and anti-phos-AKT1/2/3 (Santa Cruz Biotechnology); anti-NUP214 (Bethyl Laboratories, Inc.) Secondary antibodies were from Santa Cruz Biotechnology, Inc.

### Cell Culture

The human ovarian cancer cell lines A2780, OVCAR5 and SKOV3-ip were purchased from American Type Culture Collection and grown in recommended completed growth medium.

### IC_50_ Assay

Ovarian cancer cells were plated in 2- 24 well plates with a density of 2 × 10^4^ cell per well and were allowed to grow overnight under standard conditions. The following morning, growth medium was replaced by starving medium and the cells were allowed to grow under normal conditions. After 24 hours, the starving medium was replaced with fresh starving medium and 5μg/ ml of 20 nm AuNP was added to one of 24-well plate (sans the control wells) and returned to the incubator under normal conditions. In the following 24 hours, the starving medium was replaced with fresh starving medium and various doses of cisplatin was added to each well (ranging from 0.5 μM to 20 μM) and returned to the incubator. Following treatment, 1 μCi [^3^H]thymidine was added; 4 h later cells were washed with chilled PBS, fixed with 100% cold methanol, and collected for measurement of TCA-precipitable radioactivity. Experiments were repeated at least three separate times, with each repeat performed in triplicate. IC_50_ values were determined using GraphPad Prism. Statics were done using a two-tailed paired t-test.

### Total RNA Isolation, cDNA Synthesis and Quantitative Real-Time PCR Analysis

Total RNA was isolated from cell lines following manufacturers' instructions (Qiagen). The quality of RNA was assessed with SPECTROStar^Nano^ (BMG Labtech Inc.), and cDNA was synthesized using the Transcriptor First Strand cDNA Synthesis Kit (Roche Applied Science). Quantitative real-time PCR was conducted in triplicate for each gene of interest using SYBR Green dye and the protocol provided by Clontech. Gene expression levels were measured in an ABI PRISM 7300HT Sequence Detection System (Applied Biosystems). Relative quantification of target genes was calculated using the comparative cycle threshold (CT) method (2^−ΔΔCT^) with genes normalized to GAPDH. The sequences of the primers were listed in Table S1.

### Western Blot Analysis

Cells were lysed by RIPA buffer with proteinase inhibitors and the total proteins concentration was determined using BCA kit (Thermo Scientific). 20 μg cell lysates were electrophoresed through 4-20% gradient denaturing polyacrylamide gels (BioRad) and transferred to a polyvinylidene difluoride membrane (Millipore). The blots were probed with primary antibodies, and bound antibody was detected using enhanced chemiluminescence (Bio-Rad) according to the manufacturer's protocol. Primary antibody dilution was a 1:1000 for N-Cadherin, vimentin, IκBα, p65, NUP214, α-SMA and Akt1/2/3; 1:2000 for β-Catenin; 1:4000 for E-Cadherin; 1:20000 for β-Actin; and 1:500 for phos-Akt1/2/3. Secondary antibody dilution factors were 1:10000.

### Immunofluorescence Microscopy

Cells were grown on coverslips, washed with phosphate-buffered saline (PBS), fixed in 4% paraformaldehyde at room temperature for 15 min, washed, permeabilized for 15 min with 0.2% Triton X-100, and blocked with 3% bovine serum albumin (BSA) in PBS for 30 min at room temperature. The coverslips were incubated sequentially with appropriate primary (E-cadherin, 1:500) and secondary antibodies for fluorescence observation using a Zeiss Axiovert 200m Inverted Fluorescent Microscope.

### Side Population Assay

The side population was analyzed as previously reported [48]. Briefly, the cells were collected and suspended in DMEM with 2% FBS at the concentration of 10^6^ cells/ml. Then the cells were incubated with 5 μM DyeCycle Violet for 2 hrs at 37°C with gentle mixing at 30 min intervals. The side population cells were counted using BD LSR II Analyzer and the data were analyzed by FlowJo software.

### Fabrication of 20 nm AuNP

Citrate AuNPs were prepared as previously reported [49]. In a 250 mL flask, 2.5 mL of a 10 mM tetrachloroauric acid trihydrate (HAuCl_4_) in 90 mL water was heated to boil with vigorous stirring. Once boiling, 7.5 mL of preheated 1% sodium citrate was added rapidly. This solution was left to boil for an additional 10 min, at which point it was removed from heat and allowed to cool to room temperature while stirring. The size of the nanoparticles was determined from analysis of the dynamic light scattering (DLS) (Malvern Zetasizer Nano ZS). Zeta potential measurements were done using a clear zeta disposable capillary (Malvern DTS1061). The AuNPs were concentrated by centrifuge at 10 °C for 20 min before each use and the concentration was measured by SPECTROStar^Nano^ (BMG Labtech Inc.).

### Measurement of Gold Content by Instrumental Neutron Activation Analysis (INAA)

Samples were analyzed by INAA as previously described [12]. Briefly, samples were transferred with 100 microliters of 18 MOhm water into a pre-cleaned, high-density polyethylene irradiation vials, lyophilized to constant dry weight and mass recorded. Samples were then loaded in polyethylene transfer “rabbits” and irradiated for 90 s in a thermal flux density of ~5×10^13^ n∙cm^2^∙s^−1^.

### Measurement of Cisplatin Content by Inductively Coupled Plasma Mass Spectrometry (ICP-MS)

Uptake of cisplatin was determined by IPC-MS as [50] with modifications. The tumor fractions were digested overnight in 3 ml HNO_3_ and 1 ml H_2_O_2_. On the next day, 1 ml of aqua regia was added, and then the sample was allowed to react for another 1-2 hrs. The sample solution was then diluted to 100 mL with de-ionized water, and aqua regia (final concentration: 5%). Then the sample solution was measured by ICP-MS on a Perkin Elmer Elan 6100. Cisplatin uptake experiments were repeated 3 times, and each replicate was measured 10 times by ICP-MS. A series of cisplatin solutions were prepared before each experiment. The resulting calibration line was used to determine the amount of cisplatin in each fraction.

### Immunohistochemistry

Xenograft tumor samples were fixed in 10% formalin solution for 24 hours and transferred to 70% ethanol. Then tissues were embedded in paraffin wax according to embedding machine manufactures instructions. And 4-μm sections were prepared. Immunohistochemistry was performed according to standard protocols. Antigen retrieval was achieved by heating sections in 95 °C citrate buffer for 10 minutes. Sections were incubated with specific antibodies overnight at 4 °C. For CD31 (1:100) and Ki67 (1:100) staining, the dark brown signal was revealed after incubation with the ABC kit (Vector), followed by a diaminobenzidine (DAB) and hydrogen peroxide reaction using the DAB detection kit (Vector). Counterstaining was performed by incubating the slides in Hematoxylin for 5min. For α-SMA (1:200) staining, Alexa fluor 568-conjugated secondary antibody was used. The nuclei were visualized by incubation with DAPI, and images were examined with a fluorescent microscope. Appropriate controls were used in all cases by incubating sections with all except the primary antibodies. No staining was observed under these conditions.

### Preclinical Model of Ovarian Cancer

Female athymic nude mice (NCr-nu; 6 to 8 wks old) were purchased from the National Cancer Institute-Frederick Cancer Research and Development Center (Frederick, MD). All mice were housed and maintained under specific pathogen-free conditions in facilities approved by the American Association for Accreditation of Laboratory Animal Care and in accordance with current regulations and standards of the U.S. Department of Agriculture, U.S. Department of Health and Human Services, and NIH. All studies were approved and supervised by the Mayo Clinic Institutional Animal Care and Use Committee.

For the generation of orthotopic ovarian tumor models, SKOV3-ip cells containing luciferase were injected into the ovaries of nude mice. 4 days post tumor inoculation, tumor growth was imaged using a the Xenogen-IVIS. The mice were randomized into 4 treatment groups (n=10): (i) HBSS only, (ii) 20nm AuNP only (100 μg), (iii) Cisplatin only (500μg/kg) and (iv) Cis+AuNPs (500 μg/kg and 100μg, respectively). After randomization, mice were injected into the peritoneum with 100 μg of 20 nm citrate capped AuNPs. The AuNP treatments were thrice/week for a period of 3 weeks. Subsequent cisplatin injections were also performed thrice/week 24 hours after AuNP treatments. Mice weights were recorded weekly and their health/ behaviors were monitored daily. Efficacies of the treatment groups were compared with the control groups where mice were treated only with HBSS. After the final treatment and assessing tumor growth/ regression in these animals, mice were sacrificed by CO_2_ inhalation with tumors and tissue harvested for further analysis.

### Statistical analysis

All values are expressed as means ± SD. Statistical significance was determined using two-tailed paired *t* test between two groups. For animal experiments, 9 ~ 10 mice were assigned per treatment group. Significance between treatment groups was determined using a proposed a one-way ANOVA model using Tukey.

## SUPPORTING MATERIALS AND METHODS TABLE AND FIGURES


