# Supplementary material for: Sensitization of ovarian cancer cells to cisplatin by gold nanoparticles
Source: Oncotarget. 2014 Jul 11;5(15):6453–65. doi: 10.18632/oncotarget.2203 (PMC4171643; doi:10.18632/oncotarget.2203)
Supplement: Supplementary file 1 [file oncotarget-05-6453-s001.pdf]

Sensitization of ovarian cancer cells to cisplatin by gold nanoparticles

Supporting Materials and Methods

Chemical Reagents and Antibodies. Tetrachloroauric acid trihydrate, trisodium citrate and sodium borohydride were from Sigma-Aldrich, St. Louis, MO. [^{3}H] Thymidine was from Perkin-Elmer, (Waltham, MA). Media and PBS was purchased from Mediatech (Manassas, VA). Cisplatin was obtained from the Mayo Clinic Pharmacy services at a concentration of 50mg/ml. Scintillation cocktail was purchased through Fisher Scientific. And Alexa Fluor® 488 Phalloidin is from Life Technologies.

The following antibodies were used for Western blotting and immunofluorescence: anti–E-cadherin, anti-N-Cadherin, anti-β-Catenin, and anti-vimentin (BD Biosciences); anti-α-SMA, anti-Ki67, and anti-β-actin (Sigma-Aldrich); Anti-CD31, anti-AKT1/2/3, and anti-phos-AKT1/2/3 (Santa Cruz Biotechnology); Secondary antibodies were from Santa Cruz Biotechnology, Inc.

Cell Culture. The human ovarian cancer cell lines OVCAR5 and SKOV3-ip were grown in DMEM and McCoy's 5A medium respectively. The human ovarian cancer cell line A2780 was grown in RPMI 1640 (high glucose). All the media was supplemented with 10% fetal bovine serum and 1% antibiotics (Penicillin/Streptomycin) and the cell lines were maintained at 37°C in a humidified atmosphere consisting of 5% CO_2 and 95% air.

IC_{50} Assay. Ovarian cancer cells were plated in 2- 24 well plates with a density of 2 x 10^4 cell per well and were allowed to grow overnight under standard conditions. The following morning, growth medium was replaced by starving medium and the cells were allowed to grow under normal conditions. After 24 hours, the starving medium was replaced with fresh starving medium

and 5μg/ ml of 20 nm AuNP was added to one of 24 well plate (sans the control wells) and returned to the incubator under normal conditions. In the following 24 hours, the starving medium was replaced with fresh starving medium and various doses of cisplatin was added to each well (ranging from 0.5 μM to 20 μM; see Figure 1) and returned to the incubator. Following treatment, 1 μCi [^{3}H]thymidine was added; 4 h later cells were washed with chilled PBS, fixed with 100% cold methanol, and collected for measurement of TCA-precipitable radioactivity. Experiments were repeated at least three separate times, with each repeat performed in triplicate. IC_{50} values were determined using GraphPad Prism. Statics were done using a two-tailed paired t-test.

Total RNA Isolation, cDNA Synthesis and Quantitative Real-Time PCR Analysis. Total RNA was isolated from cell lines following manufacturers' instructions (Qiagen). The quality of RNA was assessed with SPECTROStarNano (BMG Labtech Inc.), and cDNA was synthesized using the Transcriptor First Strand cDNA Synthesis Kit (Roche Applied Science). Quantitative real-time PCR was conducted in triplicate for each gene of interest using SYBR Green dye and the protocol provided by Clontech. Gene expression levels were measured in an ABI PRISM 7300HT Sequence Detection System (Applied Biosystems). Relative quantification of target genes was calculated using the comparative cycle threshold (CT) method ($2^{-\Delta\Delta CT}$) with genes normalized to GAPDH. The sequences of the primers were listed in Table S1.

Immunofluorescence Microscopy. Cells were grown on coverslips, washed with phosphate-buffered saline (PBS), fixed in 4% paraformaldehyde at room temperature for 15 min, washed, permeabilized for 15 min with 0.2% Triton X-100, and blocked with 3% bovine serum albumin (BSA) in PBS for 30 min at room temperature. The coverslips were incubated sequentially with appropriate primary and secondary antibodies for fluorescence observation using a Zeiss Axiovert 200m Inverted Fluorescent Microscope.

Immunohistochemistry. Xenograft tumor samples were fixed in 10% formalin solution for 24 hours and transferred to 70% ethanol. Then tissues were embed in paraffin wax according to embedding machine manufactures instructions. And 4-μm sections were prepared. Immunohistochemistry was performed according to standard protocols. Antigen retrieval was achieved by heating sections in 95 ˚C citrate buffer for 10 minutes. Sections were incubated with specific antibodies overnight at 4 ˚C. For CD31 and Ki67 staining, the dark brown signal was revealed after incubation with the ABC kit (Vector), followed by a diaminobenzidine (DAB) and hydrogen peroxide reaction using the DAB detection kit (Vector). Counterstaining was performed by incubating the slides in Hematoxylin for 5min. For alpha-SMA staining, Alexa fluor 568 conjugated secondary antibody was used. The nuclei were visualized by incubation with DAPI, and images were examined with a fluorescent microscope. Appropriate controls were used in all cases by incubating sections with all except the primary antibodies. No staining was observed under these conditions.

Preclinical Model of Ovarian Cancer. Female athymic nude mice (NCr-nu; 6 to 8 wks old) were purchased from the National Cancer Institute-Frederick Cancer Research and Development Center (Frederick, MD). All mice were housed and maintained under specific pathogen-free conditions in facilities approved by the American Association for Accreditation of Laboratory Animal Care and in accordance with current regulations and standards of the U.S. Department of Agriculture, U.S. Department of Health and Human Services, and NIH. All studies were approved and supervised by the Mayo Clinic Institutional Animal Care and Use Committee.

For the generation of orthotopic ovarian tumor models, SKOV3-ip cells containing luciferase were injected into the ovaries of nude mice. 4 days post tumor inoculation, tumor growth was imaged using a the Xenogen-IVIS. The mice were randomized into 4 treatment

groups (n=10): (i) HBSS only, (ii) 20nm AuNP only (100 µg), (iii) Cisplatin only (500µg/Kg) and (iv) AuNP+Cisplatin (100 µg and 500µg/Kg, respectively). After randomization, mice were injected into the peritoneum with 100 µg of 20 nm citrate capped AuNPs. The AuNP treatments were thrice/week for a period of 3 weeks. Subsequent cisplatin injections were also performed thrice/week 24 hours after AuNP treatments. Mice weights were recorded weekly and their health/ behavior was monitored daily. Efficacies of the treatment groups were compared with the control groups where mice were treated only with HBSS. After the final treatment and assessing tumor growth/ regression in these animals, mice were sacrificed by CO_2 inhalation with tumors and tissue harvested for further analysis.

Table S1: Primers sequences used for quantitative RT-PCR

Gene Name	Sequence: 5'→3'
ABCG2	Forward: GGT CAG AGT GTG GTT TCT GTA G
	Reverse: AGA TCG ATG CCC TGC TTT AC
ALDH1	Forward: AGC CCA CAG TGT TCT CTA ATG
	Reverse: GCA GAG CAG AGG AGA TTG TTA T
CD133	Forward: ACT TGG CTC AGA CTG GTA AAT C
	Reverse: ACT CTC TCC AAC AAT CCA TTC C
CD24	Forward: CTC CTA CCC ACG CAG ATT TAT T
	Reverse: CGC CAT TTG GAT TGG GTT TAG
CD44	Forward: CAC CCA AAG AAG ACT CCC ATT C
	Reverse: GCA GTA GGC TGA AGC GTT ATA C
cKIT	Forward: CCT CGC CTC CAA GAA TTG TAT
	Reverse: CAT AGG ACC AGA CGT CAC TTT C
EpCAM	Forward: GAG ATA AAG GAG ATG GGT GAG ATG
	Reverse: AAC GAT GGA GTC CAA GTT CTG
GAPDH	Forward: GTG GTC TCC TCT GAC TTC AAC
	Reverse: CTC TCT TCC TCT TGT GCT CTT G
MDR1	Forward: GCC TAC TTG GTG GCA CAT AA
	Reverse: GTC GGG TGG GAT AGT TGA ATA C
Nannog	Forward: TCC CGG TCA AGA AAC AGA AG
	Reverse: CCT GGT GGT AGG AAG AGT AAA G
OCT4	Forward: GAG GAT CAC CCT GGG ATA TAC A
	Reverse: CTC GAT ACT GGT TCG CTT TCT C
SOX2	Forward: TTC ACA TGT CCC AGC ACT AC
	Reverse: GGA TGG GAT TGG TGT TCT CTT

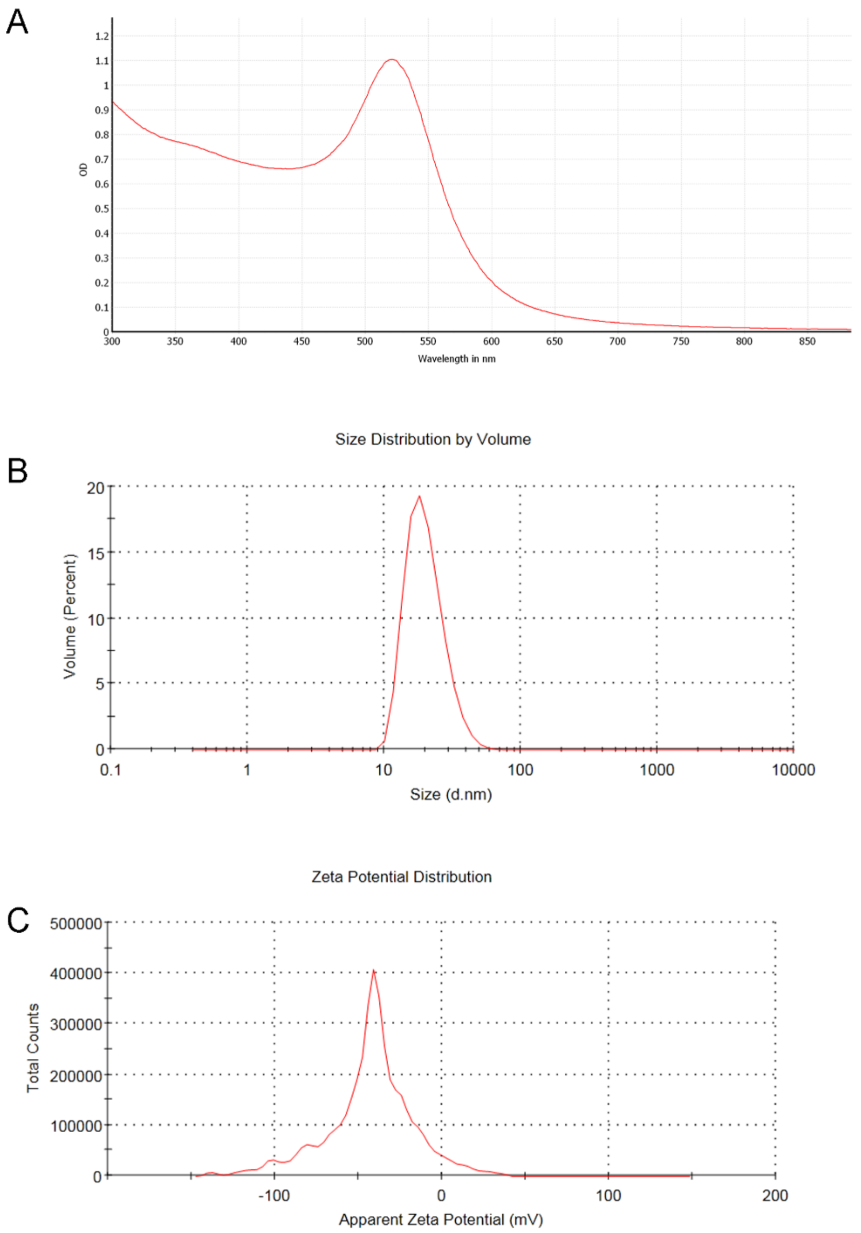

Fig. S1: Measurement of the AuNPs used in this study. (A)Representative absorption spectrum of the AuNPs used in the study. (B) DLS measurements of AuNP size. (C) Charge distribution of the functionalized AuNPs used in the study.

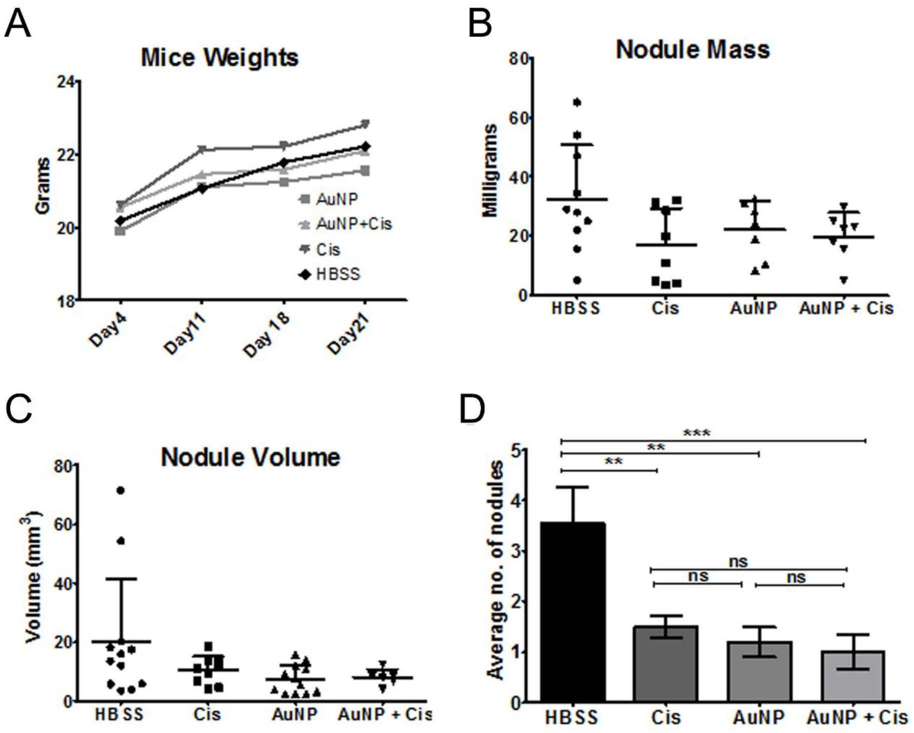

Fig. S2: (A) Mice weights were recorded during the course of the study. The increase in weight over time indicates the treatments were not toxic to the animals. (B, C) Final nodule mass (B) and volume (C) of the mice in all of the groups. The differences were not statistical significant. (D) The number of nodules in each mouse was counted and averaged. Values are means ±SD. *ns* = not significant, **P* < 0.05, ***P* < 0.01, ****P*<0.001

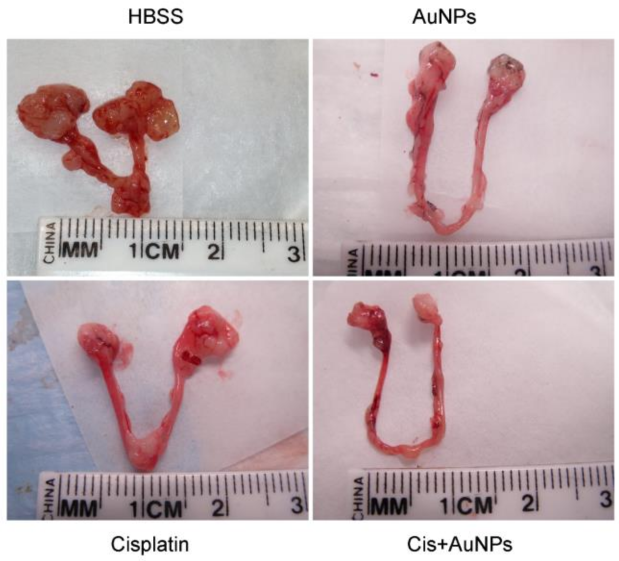

Fig. S3: Representative images of tumors from mice xenografts of SKOV3-ip cells.

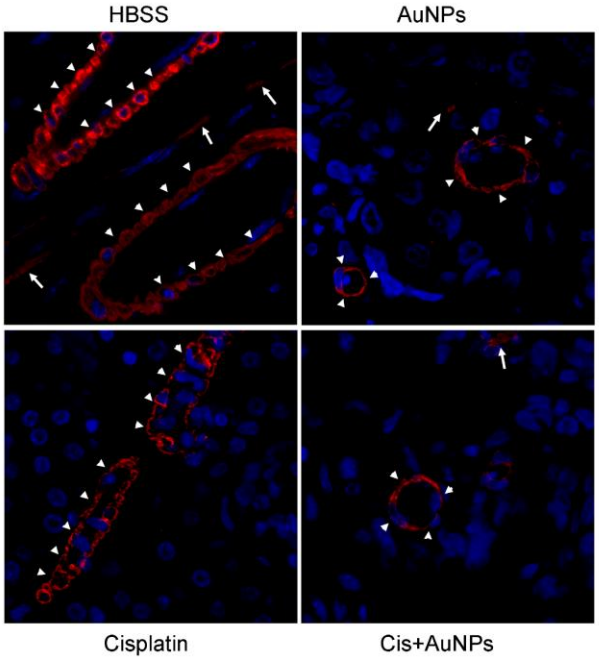

Fig. S4: Representative histology of tumors from mice xenografts of SKOV3-ip cells with positive α-SMA staining at vessel cells (Arrowhead) and myofibroblasts (Arrow).
